# Rapid validation of transcriptional enhancers using agrobacterium-mediated transient assay

**DOI:** 10.1186/s13007-019-0407-y

**Published:** 2019-03-06

**Authors:** Yuan Lin, Fanli Meng, Chao Fang, Bo Zhu, Jiming Jiang

**Affiliations:** 1 Department of Plant Biology, Michigan State University, East Lansing, MI 48824 USA; 2 Department of Horticulture, Michigan State University, East Lansing, MI 48824 USA; 3 Key Laboratory of Soybean Biology in Chinese Ministry of Education, Northeast Agricultural University, Harbin, 150030 China; 4 Department of Biological Science, College of Life Sciences, Sichuan Normal University, Chengdu, Sichuan 610101 China

**Keywords:** Enhancer validation, Luciferase, Transient assay, *Nicotiana benthamiana*

## Abstract

**Background:**

Enhancers are one of the most important classes of *cis*-regulatory elements (CREs) and play key roles in regulation of transcription in higher eukaryotes. Enhancers are difficult to identify because they lack positional constraints relative to their cognate genes. Excitingly, several recent studies showed that plant enhancers can be predicted based on their distinct features associated with open chromatin. However, experimental validation is necessary to confirm the predicted enhancer function.

**Results:**

We developed a rapid enhancer validation system based on *Nicotiana benthamiana*. A set of 12 intergenic and intronic enhancers, identified in *Arabidopsis thaliana*, were cloned into a vector containing a minimal 35S promoter and a luciferase reporter gene, and were then infiltrated into *N. benthamiana* leaves mediated by agrobacterium. The enhancer activity of each candidate was quantitatively assayed based on bioluminescence measurement. The data from this luciferase-based validation was correlated with previous data derived from transgenic assays in *A. thaliana*. In addition, the relative strength of different enhancers for driving the reporter gene can be quantitatively compared. We demonstrate that this system can also be used to map the functional activity of a candidate enhancer under different environmental conditions.

**Conclusions:**

In summary, we developed a rapid and efficient plant enhancer validation system based on a luciferase reporter and *N. benthamiana*-based leaf agroinfiltration. This system can be used for initial screening of leaf-specific enhancers and for validating candidate leaf enhancers from dicot species. It can potentially be used to examine the activity of candidate enhancers under different environmental conditions.

**Electronic supplementary material:**

The online version of this article (10.1186/s13007-019-0407-y) contains supplementary material, which is available to authorized users.

## Introduction

*Cis*-regulatory elements (CREs), which regulate gene expression, are fundamental contributors towards growth and development processes. Enhancers are one of the most common classes of CREs and are associated with the regulation of most genes in higher eukaryotes. In 1981, a 72-bp repetitive sequence element derived from the SV40 virus was the first such CRE to be described as an “enhancer”, owing to the 200-fold increase in expression of a nearby gene [1]. Since then, enhancers were found to be widely associated with transcriptional regulation in all higher eukaryotes. Several genome-wide efforts were launched to identify and characterize all enhancers in model eukaryotes [2–5]. Nevertheless, transcriptional enhancers lack positional constraint relative to their cognate genes, and can be located a few kb to several megabase (Mb) away from their target genes. Identification of enhancers associated with a specific gene has relied on prediction based on distinct chromatin characteristics associated with enhancers [5–7].

Enhancer research in plants has lagged significantly behind yeast, *Drosophila*, and mammalian species. Only a few transcriptional enhancers have been discovered in plant species [5–7]. Most of these plant enhancers were discovered due to in-depth research of the transcriptional regulation associated with specific genes. The “enhancer trapping” methodology was developed to capture functional enhancers genome-wide in several plant species [8–11]. However, the enhancer trapping methodology has several major limitations [5]. It relies on efficient production of a large number of transgenic lines, which has prevented its wide application in most plant species. For example, 31,443 independent transgenic lines were used for the enhancer trapping in rice [11]. Excitingly, a few recent studies have showed that plant enhancers, similar to those in model animal species, are associated with unique chromatin characteristics [12–14]. Since an active enhancer is associated with regulatory proteins, such as transcription factors (TFs), the genomic region associated with an active enhancer is depleted of bulk nucleosomes. These genomic regions are hypersensitive to DNase I digestion and are known as DNase I hypersensitive sites (DHSs) [15, 16]. Strikingly, 70–80% of the DHSs located in intergenic regions in *Arabidopsis thaliana* and maize (*Zea mays*) showed enhancer function [12, 14]. Therefore, DHS-based prediction and validation has opened a new venue for enhancer identification in plants.

Although enhancers can be predicted based on genome-wide datasets associated with open chromatin and other epigenomic features, the function of predicted enhancers needs to be experimentally validated. A transgenic assay using the β-glucuronidase (GUS) reporter gene has been the most popular technique for enhancer validation [9, 12]. The enhancer function may not be revealed if the transgene is inserted in a heterochromatic region in the genome. Thus, the transgenic assay requires development of multiple transgenic lines to ensure the confirmation of the true capability of each candidate enhancer in driving transcription. This can be time-consuming and technically challenging in many plant species. A protoplast-based transient transformation assay was recently developed to validate the function of predicted enhancers/promoters in maize [14]. Although this technique is more appealing than the traditional transgenic assay, it is similarly time-consuming as well as cost-prohibitive to validate a large number of candidates [14]. Thus, development of an efficient and inexpensive validation system will be essential for future enhancer research.

*Agrobacterium*-mediated transient gene expression provides a rapid and high throughput method to survey reporter gene expression [17]. This transient expression system typically uses leaf-based agroinfiltration and has been successfully applied in a number of plant species [17–20]. This transient method has also been popular for promoter analysis [21, 22]. Luciferase, first identified from firefly, is an enzyme that produces a strong bioluminescence signal [23]. Several luciferase genes were cloned from bioluminescence-producing organisms and have been widely used as bioluminescent reporters [24]. In plants, luciferase was used as a reporter as early as 1986 [25] because of its sensitivity, low background, dynamic range of emission light, and high-throughput live imaging [24, 26, 27]. These features of the luciferase reporter assay make it an attractive system to capture the dynamics of enhancer activity in real-time. Here, we developed an enhancer validation system using a luciferase reporter and *N. benthamiana*-based leaf agroinfiltration. We demonstrated that *A. thaliana* enhancers functioning in leaf tissue can be validated by this methodology. In addition, the strength of each enhancer for driving the reporter gene can be quantitatively measured and compared. This system can be used for initial screening of leaf-specific enhancers and potentially for mapping functional activity of candidate enhancers under different environmental conditions.

## Results

### Development of a luciferase-based system for enhancer validation

**Fig. 1:**
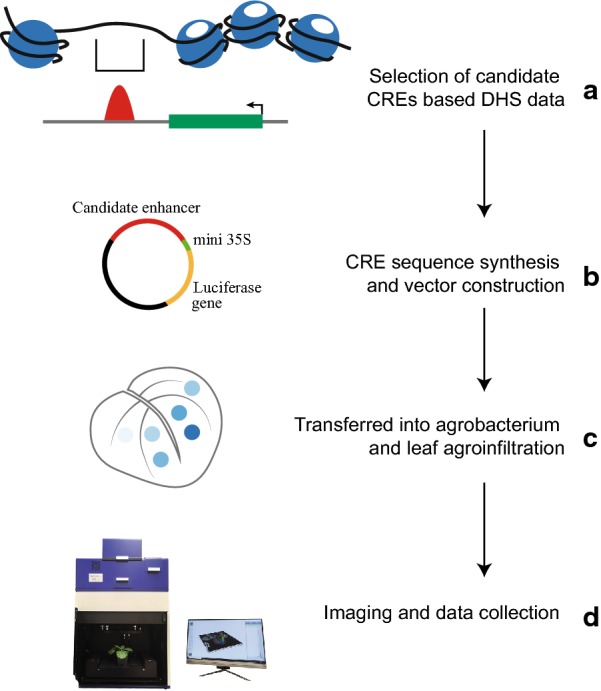
Schematic representation of the enhancer validation pipeline using a luciferase-based transient assay. **a** Candidate enhancers were predicted based on DHSs and other chromatin datasets. **b** The predicted enhancer sequence was synthesized and cloned in the pCAMBIA-CRE-LUC vector containing a mini 35S promoter and the firefly luciferase reporter gene, and was transferred into *Agrobacterium* strain GV3101. **c** Each construct was agroinfiltrated into *N. benthamiana* leaves together with both positive and negative controls. **d** Bioluminescent data were collected using the NightSHADE LB 985 plant imaging system

We developed an agrobacterium-mediated transient assay for potential enhancer activity (Fig. 1). Briefly, a candidate enhancer, typically 100 to 600 bp, which was predicted based on DHS data and other chromatin datasets [12], is synthesized and cloned into the vector pCAMBIA-CRE-LUC (Fig. 1). This vector contains a firefly luciferase reporter gene and the minimal cauliflower mosaic virus (CaMV) 35S promoter (− 50 to − 2 bp). The candidate enhancer is placed upstream of the mini35S promoter (Additional file 1: Figure S1). The transcription of the reporter gene would depend on whether the candidate enhancer is associated with enhancer function, as the mini35S promoter alone is insufficient to drive transcription of the reporter gene. This construct is then transferred into agrobacterium strain GV3101 and the bacteria are infiltrated into *N. benthamiana* leaves. A challenge of this transient assay is the variability of reporter gene expression after agroinfiltrations (Additional file 2: Figure S2). To minimized the variability our assay was based exclusively on the second extended leaf from each plant. All selected plants were 1-month old, healthy, and at the similar development stage (Additional file 2: Figure S2). We also infiltrated all the constructs in the same leaf. The bioluminescence signals derived from the construct were collected using an in vivo plant imaging system (Fig. 1).

Since the mini35S promoter lacks the necessary elements to drive expression of the luciferase gene, a construct containing only the mini35S promoter was used as a negative control in each experiment. We also developed a construct N1, containing a randomly selected genomic DNA fragment (455 bp) that is not associated with a DHS in *Arabidopsis*. N1 has no enhancer function [12] and does not generate any luciferase activity when fused to the mini35S promoter. N1 was used as the second negative control in all assays. The complete 35S promoter was used as a positive control.

### Comparative assessment of enhancers using GUS-based transgenic assay and luciferase-based transient assay

We selected a total of 12 *Arabidopsis* candidate enhancers and 6 rice DHSs for the luciferase-based transient assay. For the 12 *Arabidopsis* enhancers, the first nine (Additional file 6: Table S1) were selected from intergenic DHSs (> 1.5 kb upstream of a transcription start site or > 1.5 kb downstream of a transcription termination site) to ensure that these candidate enhancers were not associated with any promoters. The enhancer activity of these nine candidates was previously validated using permanent transformation with a GUS reporter [12]. Thus, results from the GUS-based assays can be compared to those from the luciferase-based transient assay. Three additional candidate enhancers were selected from DHSs located within introns of three different genes (Additional file 6: Table S1), and have also been assessed using GUS-based transgenic assays.

**Fig. 2:**
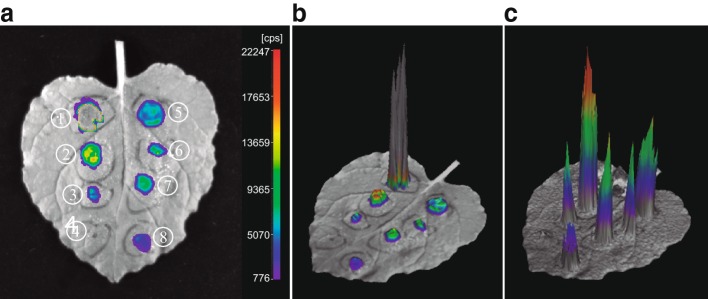
Measurement of enhancer activity based on bioluminescent imaging in vivo. **a** A representative *N. benthamiana* leaf infiltrated with constructs containing six different enhancers (sample 2, 3 and 5–8), the 35S promoter (positive control, sample1) and a mini35S promoter (negative control, sample 4). Data was collected at 30 h after agroinfiltration. Color scale of the luminescent signal intensity; purple, least intense signal; red, most intense signal; Inner gray for sample 1, over saturated intense signal. **b** Three-dimension bioluminescent signal of (A). **c** Three-dimension bioluminescent signal of (A) after excluding sample 1

All 12 constructs together with the negative and positive controls were randomly infiltrated in the same leaf from 1-month-old *N. benthamiana* plants grown in a growth chamber. Each construct was infiltrated to an ~ 1 cm^2^ region on the leaf (Fig. 2) and three leaves from different plants were used for each experiment. Bioluminescence signals derived from each construct were collected and digitized using the NightShade LB985 plant imaging system. Data were collected at 50 h after agroinfiltration.

**Fig. 3:**
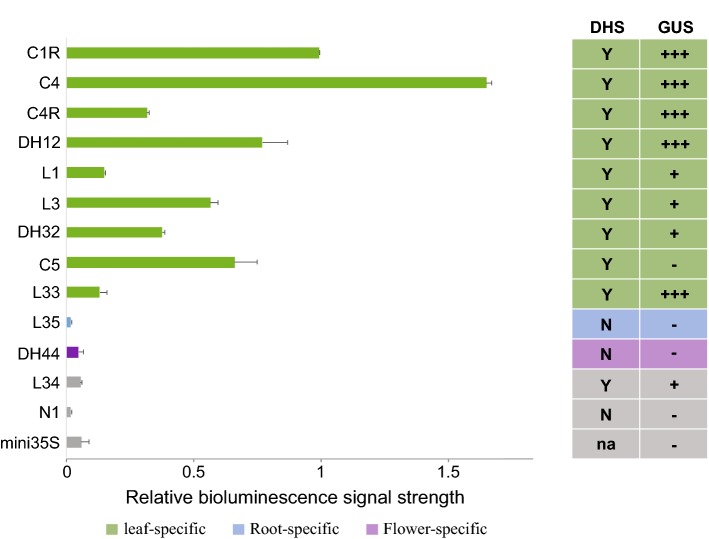
Relative signal intensity of candidate enhancers based on measurements using luciferase-based transient assays (left panel) and GUS-based transgenic assays in leaf (right panel). Left panel: Data from luciferase-based transient assays. Y-axis: 12 candidate enhancers along with two negative controls (N1 and mini35S); X-axis, relative bioluminescent signal strength normalized with enhancer C1R as standard 1. Data represent the mean ± SEM (*n* = 3). Right panel: Leaf data from GUS-based transgenic assays [12]. The strength of the enhancer in leaf is defined for three ordinal levels (+, ++, +++). No leaf GUS signal present as (−). DHS data associated with leaf tissue for each candidate enhancer is listed as yes (Y) or No (N). Color code for DHS: green, leaf-specific; blue, root-specific; purple, flower-specific; gray, not associated with DHS

The enhancer activities based on bioluminescence data from the nine intergenic DHSs were generally correlated with the data from GUS-based transgenic assays. Six constructs (C1R, C4, C4R, L1, L3 and L33) showed enhancer activities in both assays (Fig. 3). However, we observed strong bioluminescence signals from construct C5, which was negative in GUS-based transgenic assay [12] (Fig. 3). Enhancer activities were not detected in leaf tissues in both assays from the remaining two constructs (L34 and L35). We also obtained consistent results from both assays with the three constructs developed from intronic DHSs (DH12, DH32, DH44). DH12 and DH32 showed consistent enhancer activities in both assays.

### *N. Benthamiana* leaf-based transient assay can be used to validate leaf-specific enhancers

Construct L35 contained a root-specific DHS. Transgenic plants containing L35 showed strong GUS signals specifically in roots [12]. This construct did not generate any luciferase signals on *N. benthamiana* leaves in multiple assays (Fig. 3). Thus, the luciferase-based transient assay may not be feasible to examine the enhancer activity associated with CREs that are functional in roots. To further confirm the leaf-specificity of the transient assay technique, we included construct DH44 that contains a flower-specific DHS. GUS signals were observed in flower tissues in transgenic lines containing construct DH44. However, construct DH44 did not generate any luciferase signals on *N. benthamiana* leaves in multiple assays (Fig. 3).

We next investigated if leaf-specific enhancers from a monocot species can be assayed in *N. benthamiana*. We randomly selected six putative enhancers that were predicted based on previously published rice DHS datasets derived from leaf tissue [15]. Five of the six DHSs were identified in rice cultivar Nipponbare and the remaining one was identified in cultivar 9311. These DHSs were named R_DHS1 to 6, ranged from 168 to 436 bp (Additional file 6: Table S2). Transient assays revealed that three DHSs, R_DHS1 to 3, show different levels of enhancer activity, from 0.38 to 1.34 in bioluminescence signals, in *N. benthamiana* leaves (Additional file 3: Figure S3, Additional file 6: Table S3). However, the other three DHSs, R_DHS4 to 6, didn’t generate bioluminescence signals (Additional file 3: Figure S3). Thus, the *N. benthamiana* leaf-based transient assay was not consistent to evaluate putative enhancers from a monocot plant species.

### Quantitative measurements of enhancer activities using luciferase-based assay

The luciferase-based transient assay allowed us to quantify the level of enhancer activity of each candidate CRE. In addition, assays of multiple constructs on the same *N. benthamiana* leaf allowed comparative analysis of the strength of each putative enhancer (Fig. 2). In contrast, the strength of each enhancer in transgenic assays can only be arbitrarily assigned as “weak, medium, or strong” based on GUS signal strength and the percentages of transgenic plants with GUS signals in the same tissue [12]. Although GUS signals can also be quantified [28], it is a tedious procedure that can hardly be applied to a large number of samples.

Construct C1R was identified as one of the strongest enhancer in *Arabidopsis*, and was marked as “+++” in the GUS-based transgenic assay [12]. We used construct C1R as the standard “1” to compare the bioluminescence data collected from other constructs. The signal strength from construct C4, which was also marked as “+++” in the transgenic assay, was quantified as 1.65, significantly greater than C1R (*p* = 0.006, student’s *t* test) (Fig. 3). Both C4R and L33 were also marked “+++” in the transgenic assays. The signal strengths from these two constructs (0.32 and 0.13, respectively), however, were significantly lower than C1R (*p* = 1.9 × 10^−4^ and 3.3 × 10^−3^, respectively, student’s *t* test) (Fig. 3). L3 was marked as “+”, but the bioluminescence signal from L3 (0.57) was significantly stronger than L33 (0.13) (*p* = 0.028, student’s *t* test) (Fig. 3). The different relative strengths of these enhancers revealed by these two methods may be attributed to different plant species and divergence of the relevant transcription factors that bind to regulatory sequences, or by the different growing conditions in the experiments. Despite these possible variations the luciferase-based assays allow for a more accurate quantification of the strength of individual candidate enhancer and comparison of the relative strengths across different enhancers.

### Enhancers activities oscillated with light/dark cycles

**Fig. 4:**
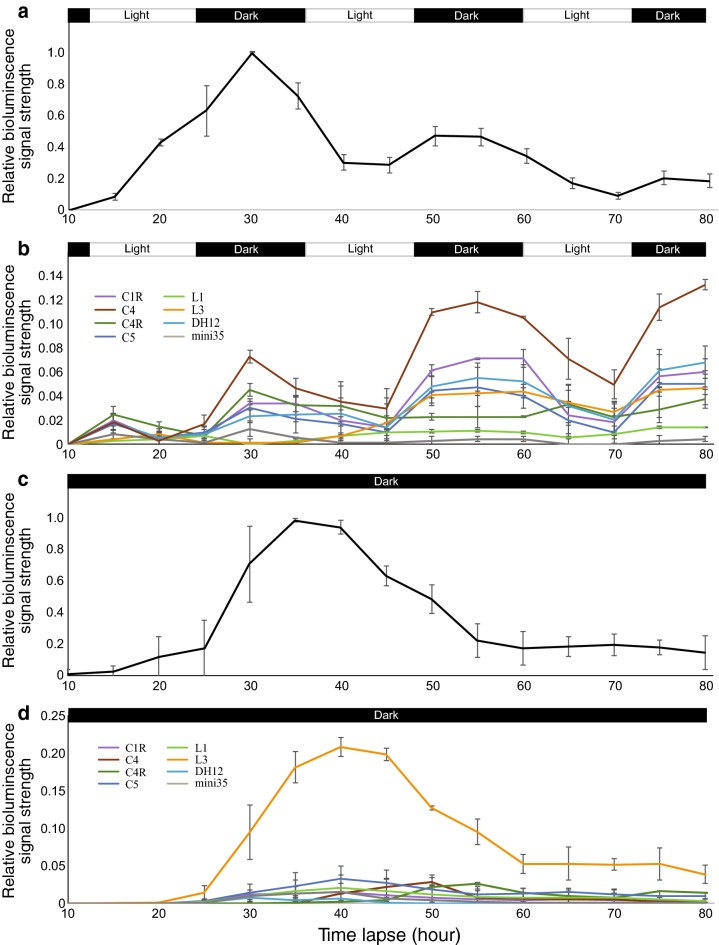
Enhancer and promoter activities associated with light/dark cycles. **a** Oscillation of the 35S promoter activity under 12 h light/12 h dark condition. **b** Oscillation of activities of seven enhancers under 12 h light/12 h dark condition. For data comparison between (**a**) and (**b**), the first peak value of the 35S promoter of (**a**) at 30 h was set as 1. **c** Luminescence activity of the 35S promoter under dark condition. **d** Luminescence activity of seven enhancers under dark condition. For data comparison between (**c**) and (**d**), the first peak value of the 35S promoter from (**c**) at 40 h was set as 1. Each data point was collected every 4 h after agroinfiltration. Data represent the mean ± SEM (*n* = 3)

Luciferase has a short half-life (high turnover rate) of just a few hours compared to GUS, which allows us to map enhancer activity at multiple developmental stages and/or under different environmental conditions. As a proof of concept, we first assayed the activity of the 35S promoter by growing *N. benthamiana* plants under regular conditions (12 h light/dark cycles, 26 °C) and collecting bioluminescence data every 5 h after agroinfiltration. Bioluminescence was detected after 10 h and the signal strength increased gradually with a peak signal at 30 h after infiltration (Fig. 4a, Additional file 6: Table S4). We observed three peaks at 30, 50 and 75 h, respectively. These peaks emerged following each of the three 12-hr light periods (Fig. 4a). A similar diurnal oscillation of the 35S promoter activity was previously reported in tobacco [29] and liverwort [30]. The limited luciferin substrate may contribute to the gradual decline of the three peaks (Fig. 4a). Interestingly, we observed only a single peak under dark condition (Fig. 4c, Additional file 6: Table S4). In addition, the peaking time under dark appeared to be delayed compared to light condition and emerged around 35 h (Fig. 4c, Additional file 6: Table S4). We compared the bioluminescence value (counts per second/cps) between the single peak under dark and the first peak under light condition. The value under dark (12.8) was significantly lower than that under light conditions (26) (*p* = 0.0053, student’s *t* test) (Additional file 4: Figure S4, Additional file 6: Table S5).

We then assessed the patterns of seven enhancers under the same conditions. Several enhancers, including C4, C1R, C5, and DH12, showed a similar activity pattern as the 35S promoter under light, with multiple peaks coinciding with the dark periods (Fig. 4b), except that a weak initial peak was observed during the first light period. Activity peaks coinciding with the dark rather than light periods could be explained by (1) detection of the bioluminescence signals is 4–6 h later after the application of a stimulus (such as light) and the exogenous substrate [24, 31]; and (2) light is required for the production of the proteins that are essential for the function of these enhancers. Thus, these proteins may not be immediately available at the early stage during light periods.

Variable activity patterns were observed for different enhancers. Enhancer C4R peaked after the first 12 h light period and then stabilized. Bioluminescence from enhancer L3 emerged after the second light period, which was clearly delayed compared to other enhancers (Fig. 4b, Additional file 5: Figure S5). The relative strength of each enhancer was well correlated with the data collected at 50 h after agroinfiltration.

Under constant dark condition the activity of most enhancers was significantly repressed compared to the activity under light condition (Fig. 4d). Interestingly, enhancer L3 showed a nearly identical activity pattern as the 35S promoter under dark conditions. A single major activity peak was observed around 40 h (Fig. 4d), which emerged earlier than the highest peak at around 55 h under light conditions (Fig. 4b). In addition, the bioluminescence value at this peak was greater under dark (2.82) compared to light exposure (1.27) (*p* = 7.7 × 10^−4^, student’s *t* test) (Additional file 6: Table S5).

## Discussion

GUS-based transgenic assay has been the traditional methodology to validate and dissect promoter or enhancer function. Transgenic lines allow analysis of candidate CREs in different tissues and different developmental stages using the transgenic plants, a key advantage of this traditional methodology. However, this transformation-based method is labor intensive and is difficult to quantitatively measure the strength of a candidate CRE [32]. Additionally, GUS staining cannot be performed on living tissue and can introduce false positives or negatives depending on the sampled development/environmental state [33]. GUS is also not suitable for the observation of conditionally or temporally regulated expression patterns due to its low turnover rate, as the half-life time (T_50_ protein) of GUS can be as high as several days [34–36].

We demonstrate that the luciferase-based transient assay has several advantages in enhancer validation compared to transgenic assays. First, this methodology is rapid. Many candidate enhancers can be synthesized and assayed in a relatively short time, which overcomes the key limiting factor of the transgenic assay. Second, luciferase activity can be measured quantitatively; thus, the relative strengths of different enhancers can be measured and compared on the same *N. benthamiana* leaf (Fig. 2). Compared to other fluorescence proteins, such as GFP, luciferase shows less auto fluorescent and no photo-bleaching in plant tissues [27]. Third, luciferase activity can yield spatiotemporal information since its T50 is only around 4 h [26, 37]. This relatively short half-life allows studies of gene expression dynamics in living cells or organisms [38]. We were able to use these characteristics of luciferase to study enhancer activities associated with light/dark cycles (Fig. 4).

Agroinfiltration can be performed only in few plant species, a key limiting factor of our methodology. Agroinfiltration is technically challenging in *Arabidopsis* [19, 39]. Although various mutation lines have been used to suppress agroinfiltration-related plant immune responses [40], the small size of *A*. *thaliana* leaves is a major obstacle for performing multi-sample detection in single leaf, which is crucial for obtaining high-throughput and replicated observations. Nevertheless, we demonstrated that *Arabidopsis* enhancers active in leaf tissue can be assayed in *N. benthamiana* plants. The function of 11 of the 12 tested *Arabidopsis* enhancers was well correlated in the two species. Only one enhancer, C5, showed no enhancer activity in transgenic assay in *Arabidopsis* but showed function in *N. benthamiana* (Fig. 3). This conflicting result may be caused by the divergence of the relevant transcription factors in the two plant species (*Arabidopsis* vs. *N. benthamiana*). Similar confliction has been reported previously, such as an enhancer from dicot species *Flaveria trinervia* behaving differently in *Nicotiana tabacum* [41]. As we predicted, the luciferase-based transient assay can be used to assay enhancers that are functional within leaves and but may not be useful to assay enhancers specific to other tissues (Fig. 3).

Transcription factors can diverged significantly between monocot and dicot species [42]. Nevertheless, some transcription factors are highly conserved among all plant species. For example, plant-specific transcription factor family GROWTH-REGULATING FACTOR (GRF) shows conserved function and DNA-binding domain to regulate leaf size in both monocot and dicot [43]. We tested six randomly selected candidate rice enhancers. Only three candidate enhancers can drive the luciferase reporter gene (Additional file 3: Figure S3). Thus, we conclude that our *N. benthamiana*-based transient assay is not reliable to validate leaf-specific enhancers from monocot species.

Many biological processes are associated with 24-h diurnal rhythms. The circadian clock is an internally (endogenously)-driven 24-h rhythm governed by a biological clock [44]. We demonstrated that most enhancers function in leaf tissue show oscillations with the daily light–dark cycles, however, the activity of these enhancers stayed low under continuous dark condition (Fig. 4). Thus, these enhancers show only a light-driven oscillation, instead of the internally (endogenously)-driven circadian clock. Since these enhancers are functional in the leaf tissue in both *Arabidopsi*s and *N. benthamiana*, the regulatory proteins essential for enhancer function may be mostly produced and accumulated under light condition. Thus, the oscillation of enhancer activity is correlated with the light-regulated production of the companion regulatory proteins of these enhancers. L3 was identified as a compound enhancer and showed functions in both leaf and root tissues in transgenic assays. In addition, its function as an enhancer is suppressed in leaf tissue but is dominant in roots [12]. Interestingly, L3 showed a distinct functional peak under dark conditions (Fig. 4d), this matches L3’s function in roots which can operate without light. Thus, the production of regulatory proteins essential for L3 function is not dependent on light. The NightShade LB985 plant imaging system (Fig. 1) allows temperature control. Thus, enhancers/promoters associated with responses to cold or heat stresses can also be validated using the current luciferase-based transient assay.

## Materials and methods

### Vector construction and agrobacteria transformation

Vector pCAMBIA1381Z-LUC [45] was modified to create a new vector: pCAMBIA-CRE-LUC, for enhancer validation. First, the mini 35S promoter was placed upstream of the luciferase gene. Then, the hygromycin resistance gene (*Hph*) (including the promoter and terminator sequences) was replaced by a reversed Nopaline synthase (NOS) promoter-NPT II-NOS terminator. This replacement was used to avoid false positive selections produced by the bidirectional 35S enhancer from the close by selection maker. With the new pCAMBIA-CRE-LUC vector, putative enhancers were individually inserted into the *Pst*I/*SpeI* restriction sites upstream of the mini promoter before being transferred into *Agrobacterium GV3101* for downstream functional validation. All plasmids were sequenced following these modifications using sequencing primers (Forward primer 5′ CAGGAAACAGCTATGAC 3′; reverse primer 5′ TCTCTTCATAGCCTTATGCAG 3′).

### Plant materials and growth condition

*N. benthamiana* seeds were sown on PRO-MIX^®^ HP MYCORRHIZAE™ soil mix (Promix, catalog number: 20381RG) and grown in a plant growth chamber (BioChamber-Enconair, GC-20) at 26 °C under a 12 h light/12 h dark cycle (150 µmol m^−2^ s^−1^ light, humidity 60%, fan speed 45%). 14 days old plants were transplanted into 3-inch pots. Assays were conducted on the second extended leaf, counting from top, from approximately 30 days old plants with 6 extended leaves. Leaves at the same developmental stage, from uniformly grown plants, and under standard light regimes, should be used to maximize reproducibility.

### Agrobacterium preparation and leaf agroinfiltration

A single colony of Agrobacterium *GV3101* was inoculated in a 15 ml tube with 5 ml of freshly prepared LB (100 μg/ml gentamicin and kanamycin). Cultures were shaken overnight at 28 °C, 250 rpm, and then 100 μl aliquots were dispensed into a 50 ml flask containing 10 ml LB with same antibiotics for approximately 12 h until the OD_600_ reached 0.5 (Spectrophotometer, Beckman, DU530). Suspensions were centrifuged at 5000×*g* for 10 min, followed by resuspension of the pellet in agroinfiltration buffer (10 mM MgCl_2_ (Ambion, AM9530G), pH 7.0, 200 µM acetosyringone (Phyto Technology Laboratories, A1104)). After 2 h at room temperature, 100 µl for each sample (allowing approximately three infiltrations) was transferred into a new tube, adding 10 µl of luciferin (Sigma, L9504) stock solution (10 mM). For each agroinfiltration, 10 μl agrobacteria contain an RNA-silencing suppressor HCPro (GeneBank accession numbers: AY775290, can improve protein expression when co-agroinfiltrated with the target stain [46]) was co-infiltrated when its OD_600_ reached 0.5. We evaluated that no potential bioluminescent signal was generated by the agrobacteria with different constructs.

Leaves were penetrated with a 27 G needle (BD, 305136), with a single infiltration for each sample, avoiding the leaf vein. A 1 ml luer-slip blunt end syringe (Thermo Scientific, 03-377-20) was used to perform the infiltration from the underside of each leaf by covering the penetration site with the syringe and carefully avoiding fully penetrating the leaf by lightly pressing a gloved finger on the other side of the leaf. A successful agroinfiltration results in the spreading of dark circled “wetting” area. The diameter of each infiltration wetting area was limited to 1–1.5 cm. Each leaf contained a mini negatives control, 35S mini positive control and C1R as the normalization standard. KINTECH lab wipes (Kimtech Science, KCC34155) were used to gently dry each infiltration spot to avoid cross contamination. The margins of each spot were outlined using a marker.

### Photon-counting experiments and data collection

The NightSHADE LB 985 (Berthold Technologies USA) in vivo plant imaging system was employed to detect bioluminescent signals. Up to four live plants following agroinfiltration can be placed in the NightSHADE instrument at the same time. After agroinfiltration, plants were kept in the NightSHADE LB 985 under dark for 12 h. For all data collection, luminescence signals were collected with 40 s scanning. For time lapse tracking, plants were placed in the NightSHADE LB 985 chamber following agroinfiltration, with the temperature set to 27 °C using a refrigerated/heating circulating system (Huber, Ministat 125). Plants were left overnight for 12 h under dark conditions before being subjected to a 12 h light/12 h dark cycle. After 11 h of darkness (1 h prior to light cycling) bioluminescent signal data was collected every 4 h. Plants were watered every 2–3 days. Data were analyzed by the IndiGO™ software. The average luminescence signal for each sample was collected (cps, count per second), and enhancer C1R was employed as the normalization standard as 1. Three independent replicates were used for each analysis.

Notes: (a) Plant transpiration increases the chamber humidity, especially during the daytime. To compensate for this we set the chamber temperature as 27 °C, which is higher than the traditional room temperature of 25 °C. This can help reduce the chamber condensation caused by plant transpiration. Absorbent tower papers can be used if condensation is found inside the chamber; (b) The NightSHADE LB 985 does not have an autofocus lens, and light cycling will result in cyclic vertical leaf movements that can cause miss-focusing in addition to a weakened bioluminescent signal. To address this we simply used twisted paper clips to stabilize agroinfiltrated leaves.

## Additional files

**Additional file 1: Figure S1.** Enhancer validation vector map. An enhancer candidate is inserted into the PstI/SpeI restriction sites upstream of the mini promoter.

**Additional file 2: Figure S2.** Variability of the luciferase-based transient assay in *N. benthamiana* leaf. The 35S promoter was used to test the variability of different leaves (a, b), different plants (c), and different dates (d, e). Data represent the mean ± SEM (*n* = 3).

**Additional file 3: Figure S3.** Relative signal intensity of six rice DHS based on luciferase-based transient assays. Y-axis: six randomly selected DHS from rice cultivars along with negative control (mini35S); X-axis, relative bioluminescent signal strength normalized with enhancer C1R as 1. Data represent the mean ± SEM (*n* = 3).

**Additional file 4: Figure S4.** Bioluminescent signal intensity of 35S promoter and L3 enhancer under different light/dark condition. Y-axis: 35S (Black) and L3 (Orange) enhancers. Light: 12 h light/12 h dark cycle, Dark: continuous dark without light; X-axis, bioluminescent signal identity (counts per second). Data represent the mean ± SEM (*n* = 3) (**p < 0.01, Student’s t test).

**Additional file 5: Figure S5.** Different patterns of enhancer and promoter activities associated with light/dark cycles. (**A**) Oscillation of activities of enhancers C1R, C4, C5 and MFL under 12 h light/12 h dark condition. (**B**) Oscillation of L3 enhancer. (**C**) Oscillation of activities of C4R enhancer. Data represent the mean ± SEM (*n* = 3).

**Additional file 6.** Supplemental Tables 1–5.
